# Structure-based molecular characterization and regulatory mechanism of the LftR transcription factor from *Listeria monocytogenes*: Conformational flexibilities and a ligand-induced regulatory mechanism

**DOI:** 10.1371/journal.pone.0215017

**Published:** 2019-04-10

**Authors:** Choongdeok Lee, Meong Il Kim, Jaewan Park, Minsun Hong

**Affiliations:** Division of Biological Science and Technology, Yonsei University, Wonju, Republic of Korea; University of Manchester, UNITED KINGDOM

## Abstract

*Listeria monocytogenes* is a foodborne pathogen that causes listeriosis and can lead to serious clinical problems, such as sepsis and meningitis, in immunocompromised patients and neonates. Due to a growing number of antibiotic-resistant *L*. *monocytogenes* strains, listeriosis can steadily become refractory to antibiotic treatment. To develop novel therapeutics against listeriosis, the drug resistance mechanism of *L*. *monocytogenes* needs to be determined. The transcription factor LftR from *L*. *monocytogenes* regulates the expression of a putative multidrug resistance transporter, LieAB, and belongs to the PadR-2 subfamily of the PadR family. Despite the functional significance of LftR, our molecular understanding of the transcriptional regulatory mechanism for LftR and even for the PadR-2 subfamily is highly limited. Here, we report the crystal structure of LftR, which forms a dimer and protrudes two winged helix-turn-helix motifs for DNA recognition. Structure-based mutational and comparative analyses showed that LftR interacts with operator DNA through a LftR-specific mode as well as a common mechanism used by the PadR family. Moreover, the LftR dimer harbors one intersubunit cavity in the center of the dimeric structure as a putative ligand-binding site. Finally, conformational flexibilities in the LftR dimer and in the cavity suggest that a ligand-induced regulatory mechanism would be used by the LftR transcription factor.

## Introduction

*Listeria monocytogenes* is a gram-positive bacillus that is responsible for listeriosis in humans. Listeriosis occurs by gastrointestinal ingestion of food contaminated by *L*. *monocytogenes* [1]. *L*. *monocytogenes* is able to penetrate the mucosal membranes of the intestine, brain barrier, and placenta. Therefore, *L*. *monocytogenes* can cause serious problems, such as sepsis, meningitis, and even death, in elderly and immunocompromised individuals, and may induce miscarriage or stillbirth in pregnant women [2–6]. In particular, because *L*. *monocytogenes* can grow at low temperature, extra caution is required for food storage even during refrigeration [7]. Moreover, since the number of antibiotic-resistant *L*. *monocytogenes* strains is increasing [8], listeriosis has steadily become refractory to antibiotic treatment. Therefore, it is necessary to obtain in-depth knowledge about the drug resistance mechanism of *L*. *monocytogenes* and to develop novel antibiotics against listeriosis.

The *lftR* gene that encodes a *Listeria* protein facilitating invasion/transcriptional regulator was first identified in 2015 and is commonly found in diverse *L*. *monocytogenes* strains [9]. The LftR protein transcriptionally regulates the expression of the *lmo0980* gene, which encodes a putative multidrug resistance transporter, LieAB (Listerial importer of ethidium bromide as artificial substrate), and facilitates the export of toxic chemicals from the cytoplasm to the extracellular space [9]. In addition, deletion of the *lftR* gene decreases the infection rate in host cells, implicating that LftR is a key virulence factor in *L*. *monocytogenes*-mediated pathogenicity.

Based on an analysis of primary amino acid sequences, LftR belongs to the phenolic acid decarboxylase regulator (PadR) family. In various bacteria, including *Bacillus subtilis*, *L*. *monocytogenes*, *Pediococcus pentosaceus*, *Lactococcus lactis*, and *Vibrio cholera* [10–14], PadR family members were shown to enhance bacterial survival. In the presence of toxic compounds, such as phenolic acids and antibiotic chemicals, PadR family proteins directly sense toxic substances and transcriptionally upregulate the expression of detoxifying enzymes or efflux pumps to lower the intracellular concentration of detrimental chemicals. As transcriptional regulators, PadR family members commonly contain a winged helix-turn-helix (wHTH) motif to interact with operator DNA.

Due to intrinsic variations in sizes and tertiary structures, the PadR family is further divided into two subfamilies, PadR-1 and PadR-2, which consists of ~200 and ~100 residues, respectively. PadR-1 subfamily proteins contain two structurally and functionally separated domains, namely an N-terminal wHTH domain for DNA interaction and a C-terminal helical domain for dimerization. The structural study of a PadR-1 member from *B*. *subtilis* (BsPadR) demonstrated that each subunit of the BsPadR dimer accommodates one ligand molecule in an intramolecular pocket between the N-terminal and the C-terminal domains, resulting in a molecular stoichiometry of 2:2 [15]. Unlike the PadR-1 subfamily, the PadR-2 subfamily adopts a single-domain structure and forms a dimer using the helices located before and after the wHTH motif. A member of the PadR-2 subfamily, LmrR from *L*. *lactis*, was structurally resolved to accommodate one ligand molecule into an intersubunit pocket of an LmrR dimer [16–18]. The comparative analysis of the BsPadR-DNA and BsPadR-ligand structures demonstrated that a PadR-1 subfamily member mediates transcriptional regulation through ligand-induced allostery [15]. However, our understanding of the transcription regulatory mechanism of the PadR-2 subfamily is highly limited due to the lack of information about the operator DNA binding mode of PadR-2 subfamily members.

LftR contains 108 residues and is expected to belong to the PadR-2 subfamily. To provide a molecular insight into the operator DNA recognition mechanism of the PadR-2 subfamily, we determined the crystal structure of LftR and performed a structure-based mutagenesis analysis of LftR. As in the PadR-1 subfamily, two conserved tyrosine residues in the wHTH motif play a key role in DNA recognition by LftR. In addition, a unique DNA-binding site (K9) that was not observed in the PadR-1 subfamily was identified in LftR. Thus, we propose that LftR employs a conserved but diverged DNA recognition mechanism compared to the PadR-1 subfamily. Our biophysicochemical and comparative analysis of the PadR-2 member from the pathogenic bacteria enhance our understanding of the antibiotic resistance mechanism used by *L*. *monocytogenes*.

## Results and discussion

### Crystal structure of LftR

To prepare LftR protein for structural and biochemical studies, LftR protein was recombinantly expressed in two forms, one containing an N-terminal His_6_ affinity tag (LftR_NtH_) and the other with a C-terminal affinity tag (LftR_CtH_), using *E*. *coli* cells (S1A Fig). The LftR_NtH_ protein was treated with thrombin protease to remove the N-terminal His_6_ tag, and the tag-free LftR protein (LftR_ΔNtH_) was used for our molecular studies (S1B Fig). The LftR_CtH_ protein was purified with the C-terminal His_6_ tag. To analyze the oligomerization state of LftR, each of the purified recombinant LftR proteins (LftR_ΔNtH_ and LftR_CtH_) was loaded on a gel-filtration chromatography column (S1C Fig). The apparent molecular weights of both LftR proteins were estimated to be 20–24 kDa, whereas the calculated molecular weights are ~12 kDa, indicating that LftR forms a homodimer in solution regardless of construct designs.

As a first step in the understanding of LftR at a molecular level, LftR_ΔNtH_ and LftR_CtH_ structures were determined and refined at 2.3 and 2.8 Å resolutions, respectively (S1 Table and S2 Fig). Although the LftR_ΔNtH_ and the LftR_CtH_ structures belong to different space groups (P3_2_21 and P6_4_, respectively), their overall structures are similar and thus the LftR_ΔNtH_ structure will be used to describe the structural features of LftR unless otherwise specified.

**Fig 1:**
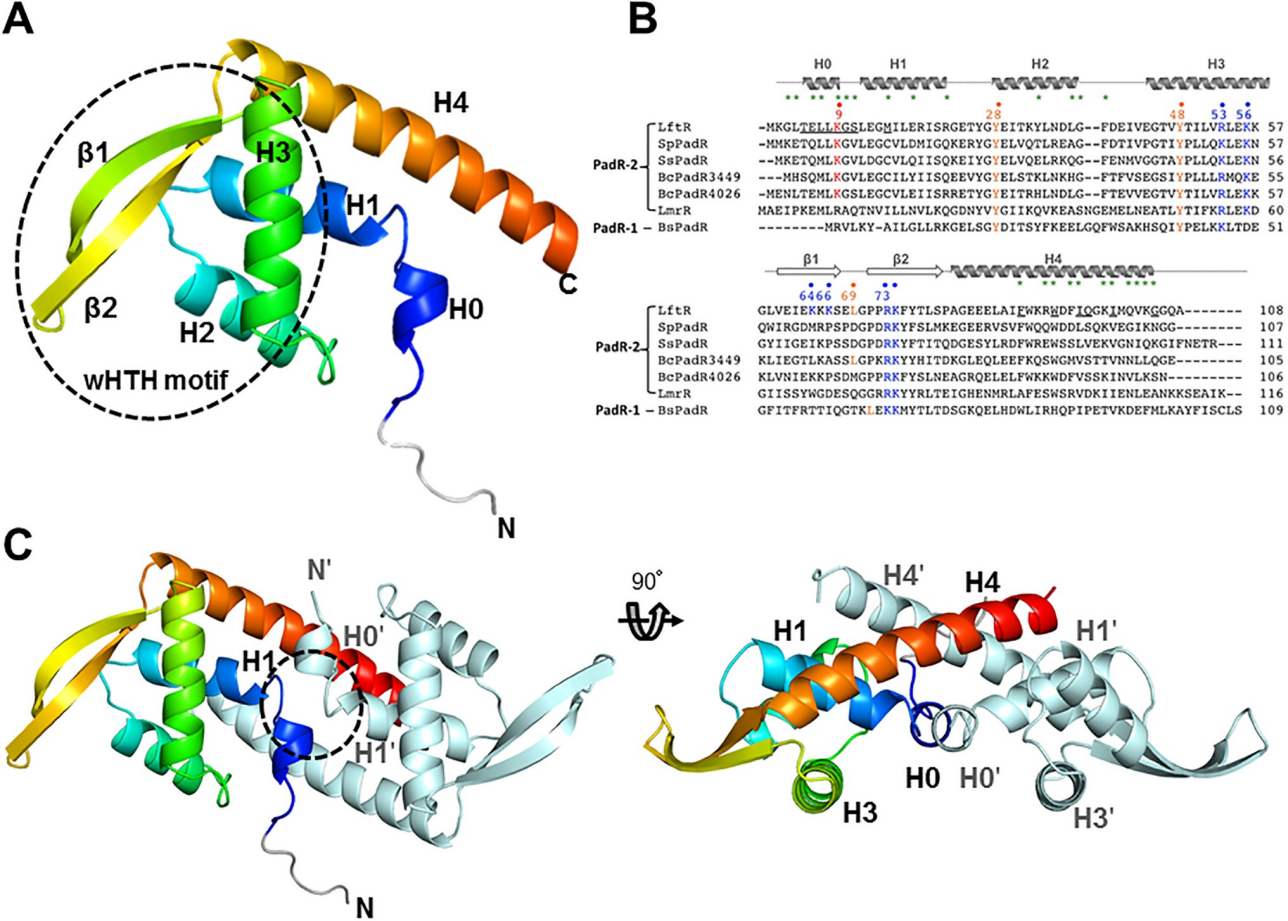
LftR crystal structure and its sequence alignment with PadR-1 and PadR-2 subfamily members. (A) Monomeric structure of LftR in rainbow-colored ribbons (N-terminus, blue; C-terminus, red). The N-terminal and C-terminal ends of LftR are labeled as “N” and “C,” respectively. Secondary structure elements are labeled (H, helix; β, β-strand). The wHTH motif contains the H2 and H3 helices, the β1 and β2 strands, and their connecting loops. The non-LftR residues that were introduced to design the recombinant protein expression construct are in grey. (B) Sequence alignment of LftR homologs. The secondary structures of LftR are shown above the LftR sequence. The LftR residues involved in dimerization are highlighted with green stars. The LftR residues that participate in DNA binding are highlighted by dots and labeled with their residue numbers above the sequences (LftR-specific DNA-binding residue, K9, red; PadR family-conserved residues, orange; positively charged patch residues of LftR, blue). The LftR residues that sterically clash with ethidium bromide in the complex model of LftR and ethidium bromide are underlined. (C) The dimeric structure of LftR is shown as ribbons (rainbow and light cyan). The intersubunit cavity of the LftR dimer is delineated by a dotted circle in the left figure.

The asymmetric unit of the LftR_ΔNtH_ crystal contains two subunits, namely A (residues from -5 to 105; the six N-terminal residues, from -5 to 0, correspond to additional residues that originate from the cloning vector) and B (residues from 3 to 108), which form a homodimer in an isosceles triangle shape (Fig 1). Each LftR subunit consists of five α-helices (H0-H4) and two β-strands (β1 and β2) with a topology of H0 (residues 4–8)–H1 (residues 11–22)–H2 (residues 27–37)–H3 (residues 44–57)–β1 (residues 60–66)–β2 (residues 70–78)–H4 (residues 80–104) (Fig 1A and 1B). The H2 and H3 helices form the wHTH motif together with a β-stranded wing containing the β1 and β2 strands.

**Fig 2:**
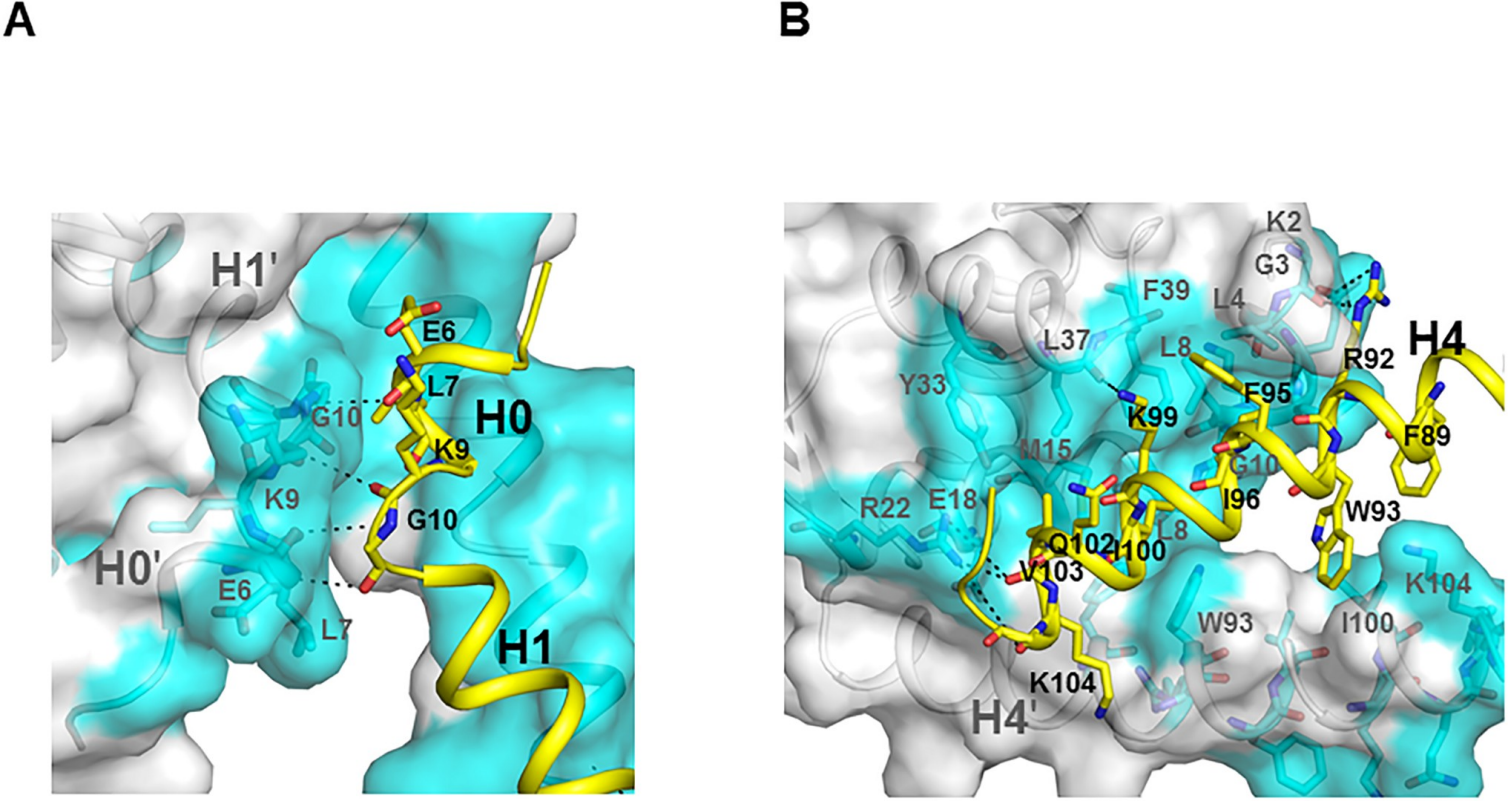
Dimerization contacts of LftR. One LftR subunit is shown as gray ribbons with surface representation, and its dimerization interface residues are highlighted by cyan surfaces and sticks. The other LftR subunit is shown as yellow ribbons, and its dimerization interface residues are depicted as yellow sticks. Hydrogen bonds are represented by dotted lines. (A) Dimerization interface at the N-terminal region of LftR. (B) Dimerization interface generated by the LftR H4 helix.

LftR dimerization occurs using 25 residues (buried surface area, ~3380 Å^2^) from the H0, H1, H2, and H4 helices and the H0-H1 loop through numerous intersubunit interactions (Figs 1C and 2). The H4 and H4ʹ helices (the prime symbol denotes the dimerization partner) form the two identical faces of the isosceles triangle of the LftR dimer, and the H3 and H3ʹ helices and the winged regions are arranged into the bottom face. In the LftR dimerization interface, the main chains of two subunits at the E6 and L7 residues of the H0 helix and the K9 and G10 residues of the H0-H1 loop run antiparallel and generate a symmetrical intersubunit H-bond network between carbonyl oxygen atoms and amide nitrogen atoms (Fig 2A). In addition to the LftR N-terminal residue interactions, the H4 helix mediates numerous intersubunit interactions with the H0ʹ, H1ʹ, H2ʹ, and H4ʹ helices for LftR dimerization (Fig 2B). The H4 and H4ʹ helices run across the middle of the helices and form hydrophobic interactions using five apolar residues (F89, W93, I96, and I100, and V103). The C-terminal residues (I100, V103, K104, and G105) of the H4 helix make van der Waals contacts and H-bonds with the H1ʹ helix residues (M15ʹ, E18ʹ, and R22ʹ). Moreover, the H4 helix (R92, W93, F95, I96, K99, I100, Q102, and V103) stabilizes the LftR dimer by interacting with the H0ʹ and the H2ʹ helices (K2ʹ, G3ʹ, L4ʹ, Y33ʹ, L37ʹ, G38ʹ, and F39ʹ). In particular, the nitrogen atoms of the R92 and K99 sidechains at the H4 helix create H-bonds with the carbonyl oxygen atoms of K2ʹ and G3ʹ residues at the H0ʹ helix and those of the L37ʹ residue at the H2ʹ helix, respectively. Therefore, the H0, H1, H2, and H4 helices and the H0-H1 loop of LftR are required for LftR dimerization.

### DNA-binding ability of LftR as a transcription factor

**Fig 3:**
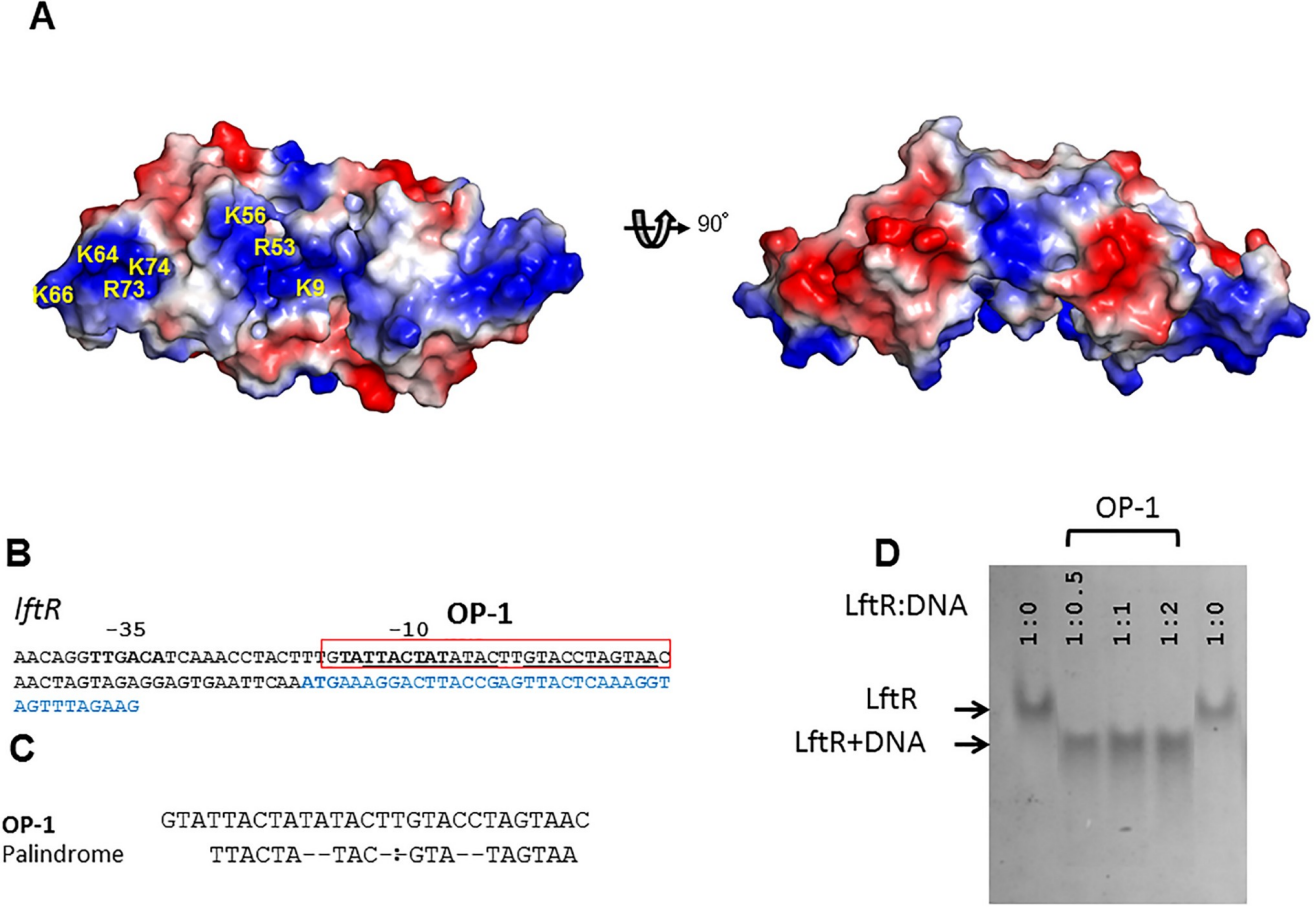
Operator DNA recognition by LftR. (A) Electrostatic surface potentials of the LftR dimer (negative charge, red; positive charge, blue). A positive patch is observed on the bottom of the LftR dimer structure, and its positively charged residues are labeled in one LftR subunit (left). The orientation of the left figure is identical to the left figure in Fig 1C. (B) Putative operator DNA sequence (OP-1) of LftR located upstream of the LftR-encoding region (light blue). (C) Sequence analysis of the OP-1 operator site. Palindrome site sequences are indicated under the OP-1 sequence. (D) Analysis by electrophoretic mobility shift assay (EMSA) of the interaction between LftR and the operator DNA (OP-1). For the EMSA, a constant amount of LftR protein (3 μg, ~240 pmol) was incubated with operator DNA at different molar ratios indicated above the gel. The gel was stained with Coomassie brilliant blue to visualize protein bands. An uncropped gel image is shown in S3 Fig.

**Fig 4:**
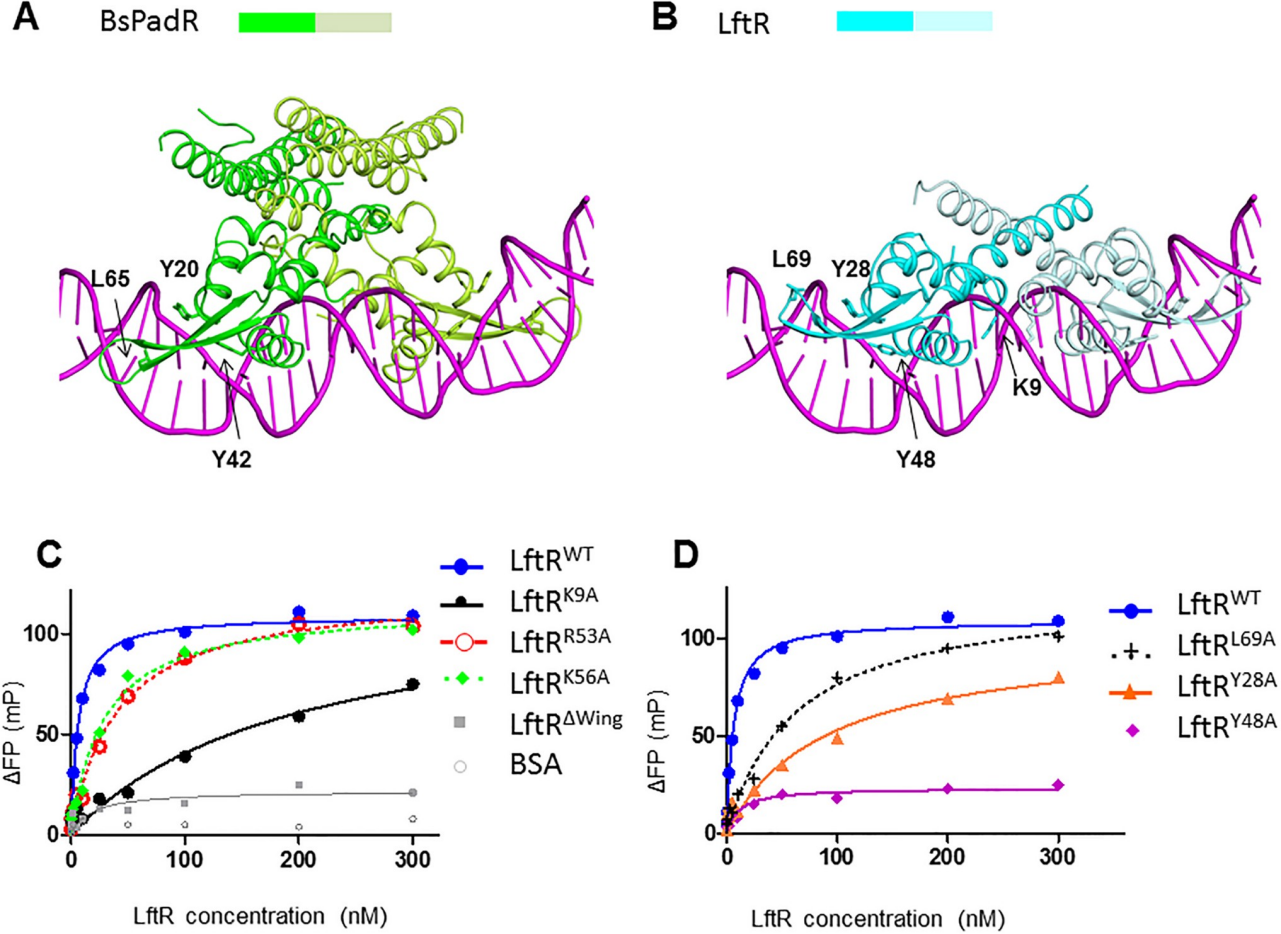
DNA recognition mode of LftR in comparison with BsPadR. (A) Structure of a complex between BsPadR (green and light green) and its operator DNA (magenta). The Y20, Y42, and L65 residues of BsPadR were shown to be critical in DNA binding and are represented by sticks with labels in the figure. (B) Homology-based structural model of the LftR-DNA complex (LftR, cyan and light cyan; DNA, magenta). The LftR Y28, Y48, and L69 residues that correspond to BsPadR Y20, Y42, and L65, respectively, are shown as sticks with labels. The LftR-specific DNA-binding residue K9 is also depicted with sticks. (C, D) Fluorescent polarization (FP) analysis for the interactions of wildtype and mutant LftR proteins with 28-mer OP-1 dsDNA.

LftR belongs to the PadR-2 subfamily and was proposed to be a transcription factor for the *lmo0980* gene that encodes a putative multidrug transporter [9]. To be consistent with the deduced function of LftR as a transcription factor, an electrostatic potential surface analysis of the LftR structure located a positive patch (K9, R53, K56, K64, K66, R73, and K74), which could facilitate DNA binding by chemically stabilizing the negatively charged phosphate groups of DNA (Fig 3A). To address whether LftR directly interacts with DNA, an electrophoretic mobility shift assay (EMSA) and a fluorescent polarization (FP) assay were performed using the purified LftR protein and the partially palindromic dsDNA of a tentative operator site. One operator site of LftR, OP-1, was identified in the promoter regions of the *lftR* using BPROM software and by manual searches (Fig 3B and 3C). The OP-1 site contains partially palindromic sequences (Fig 3C). In the EMSA, the OP-1 dsDNA altered the mobility of the LftR protein (Fig 3D and S3 Fig), indicating their direct interaction. In the FP assay, LftR displayed a specific binding curve in the presence of OP-1 dsDNA with an equilibrium dissociation constant (K_d_) of 6.2 ± 1.5 nM (Fig 4C and Table 1). These results suggest that LftR functions as a transcription factor presumably to regulate the transcriptional expression of its own gene.

**Table 1:** Operator dsDNA-binding analysis of LftR wildtype and mutants.

LftR proteins	K_d_ (nM)	K_d_ ratio (mutant/wildtype)
Wildtype	6.2 ± 1.5	1.0
K9A	197.0 ± 12.1	31.7
Y28A	89.6 ± 1.9	14.5
Y48A	>1,000	>1,000
R53A	40.1 ± 0.8	6.5
K56A	28.7 ± 2.3	4.6
L69A	62.6 ± 1.3	10.1
ΔWing	>1,000	>1,000

The dsDNA-binding affinity of LftR was determined by fluorescent polarization assay and is represented by the dissociation equilibrium constant. The data represent the mean ± S.D. from at least three independent experiments.

To reveal the operator DNA-binding mode of LftR, homology-based structural modeling of the LftR-DNA complex was performed using the structure of a complex between BsPadR and dsDNA [15] as a template (Fig 4 and S4A and S4B Fig). The monomer structures of LftR and BsPadR were superimposed using LftR residues 10–100 and BsPadR residues 1–90 (root mean square deviation, 1.32 Å for 77 Cα atoms with a sequence identity of 27%). Then, the DNA molecules from the PadR-DNA structure [15] were merged with the LftR structure to generate a LftR-DNA complex model. In the complex model, the seven positively charged residues of LftR in the positive patch interact with negatively charged phosphate groups of DNA, and six of them, except for the K9 residue, are located at the wHTH motif. In other wHTH-containing transcription factors, such as BsPadR and OhrR [15, 19], wHTH motif is critical in DNA binding. Indeed, the individual replacement of the positively charged residues, R53 and K56, with an alanine residue lowered the DNA-binding affinity of LftR by 4-6-fold (Fig 4C and S5 Fig and Table 1). Moreover, deletion of the winged region (residues 59–78, including K64, K66, R73, and K74 residues) in LftR abrogated DNA binding (LftR^ΔWing^ mutant in Fig 4C). Therefore, the positively charged residues of LftR in the wHTH motif are undoubtedly engaged in operator DNA recognition as observed in other wHTH transcription factors.

### Similar DNA recognition modes of LftR and PadR

The Y20, Y42, and L65 residues of BsPadR in the wHTH motif were shown to play a key role in operator DNA binding, and the two tyrosine residues were proposed to be the signature residues of the PadR family due to their high sequence conservation in both PadR-1 and the PadR-2 subfamily members (Fig 4A) [15]. The three residues are also found in LftR as Y28, Y48, and L69. These residues interact with phosphate backbones of DNA in the LftR-DNA model in a similar mode to the equivalent residues of BsPadR in the BsPadR-DNA structure (Fig 4B). To verify the requirement of Y28, Y48, and L69 residues in LftR for DNA interaction, the three residues were individually mutated to alanine, and the OP-1 DNA-binding affinities of the alanine mutants (LftR^Y28A^, LftR^Y48A^, and LftR^L69A^) were determined by the FP assay (Fig 4D and S5 Fig). Alanine mutations at the conserved tyrosine residues, Y28 and Y48, decreased DNA-binding affinity by ~14-fold and at least 1000-fold, respectively (Fig 4D and Table 1). Moreover, the LftR^L69A^ mutant displayed ~10-fold lower DNA-binding affinity than the LftR wildtype (LftR^WT^). Thus, the Y28, Y48, and L69 residues of LftR play a key role in DNA binding, suggesting that LftR uses two signature tyrosine residues (Y28 and Y48) of the PadR family as well as a leucine residue (L69) of the winged region to recognize the major and minor grooves of operator dsDNA, respectively, as in PadR-1 subfamily members such as PadR and VanR.

### Unique DNA-binding mode of LftR through the K9 residue

Among the seven positively charged residues in the positive patch of the LftR structure, the K9 residue does not belong to the wHTH motif and instead is found at the H0-H1 loop. Noticeably, the K9 residue made a structural clash with DNA at the minor groove of dsDNA in the LftR-DNA complex model, suggesting that the LftR K9 residue would reposition its side chain to satisfy shape and chemical complementarity with DNA molecules and adjacent LftR residues. To examine if the K9 side chain is required for DNA interaction, the K9 residue was replaced with alanine to generate the LftR^K9A^ mutant. LftR^K9A^ exhibited an equilibrium dissociation constant of 197.0 ± 12.1 nM, indicating ~32-fold lower DNA-binding affinity than the LftR^WT^ (Fig 4D and Table 1). The critical role of the K9 residue in DNA recognition seems to be unique to LftR because the BsPadR R2 residue that is equivalent to the LftR K9 residue is not involved in a minor groove interaction, and its mutation to alanine did not change the DNA-binding affinity of BsPadR [15]. Based on the comparative structural and mutational analyses, we propose that a DNA recognition mode for LftR would be diverged from that of BsPadR at and near the LftR K9 residue.

### Conformational diversity of LftR

**Fig 5:**
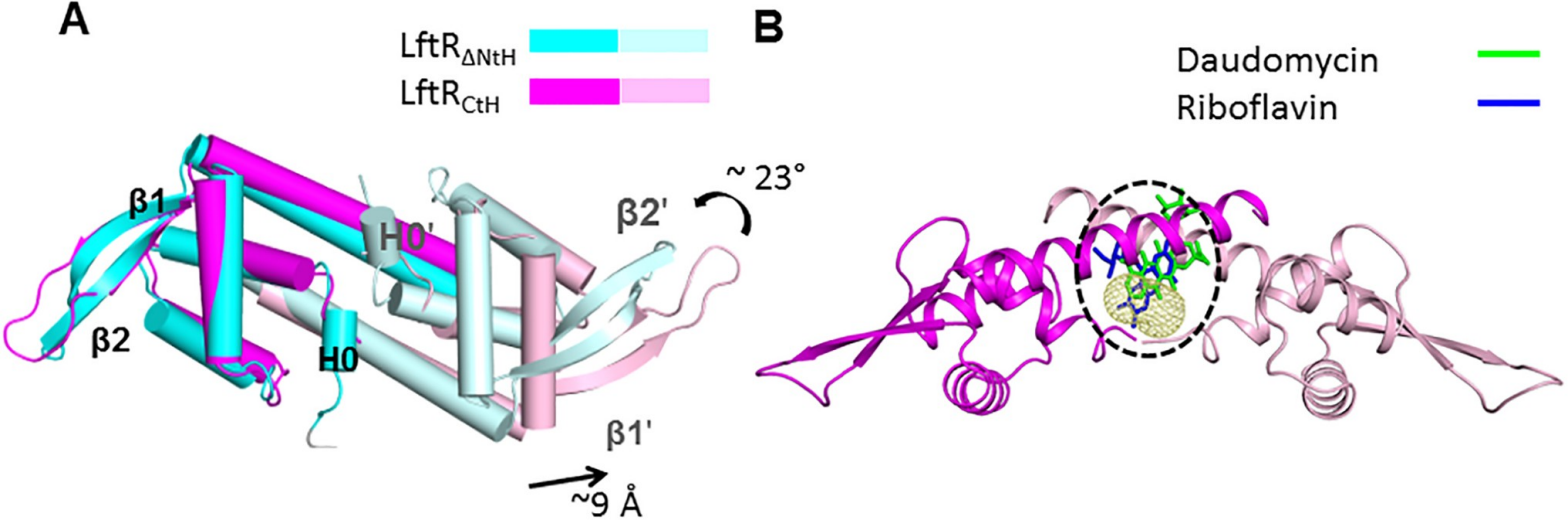
Structural diversity and intersubunit cavity of LftR. (A) The LftR_ΔNtH_ structure (cyan and light cyan) was superimposed on the LftR_CtH_ structure (magenta and light magenta) using the wHTH motifs in one subunit. (B) Intersubunit cavity located in the center of the LftR_CtH_ dimer structure. The structure of the LftR_CtH_ dimer is shown as magenta and light magenta ribbons. The intersubunit cavity of LftR is represented by yellow mesh and highlighted by a dashed circle. The structures of the LmrR-ligand complexes (LmrR-riboflavin, PDB accession code 4ZZD; LmrR-daunomycin, PDB accession code 3F8F) were overlaid on the LftR structure, and the ligand molecules of LmrR are shown as sticks (riboflavin, blue; daunomycin, green) with the LftR_CtH_ dimer.

Previous structural studies of bacterial transcription factors have demonstrated that transcription factors adopt diverse conformations depending on binding partners and environments [15, 20, 21]. A comparative analysis of the LftR_ΔNtH_ and LftR_CtH_ structures highlights the conformational diversity of LftR. The monomeric structures of LftR_ΔNtH_ and LftR_CtH_ are mostly similar, but local structural differences are observed at the H0 helix and the β1 and β2 strands (Fig 5A). Residues 4–8 form an α-helix (H0 helix) in the LftR_ΔNtH_, whereas the corresponding region of LftR_CtH_ adopts an extended coil structure, indicating the flexible nature of the LftR N-terminal residues. In addition, the β1 and the β2 strands in the wHTH motif of the LftR_ΔNtH_ (β1, residues 60–66; β2, residues 70–78) are longer than those of the LftR_CtH_ structure (β1, residues 61–65; β2, residues 73–76). Thus, the LftR_CtH_ structure exhibits a longer loop (residues 66–72) between the β1 and β2 strands than the LftR_ΔNtH_ structure (residues 67–69).

In addition to the local structural differences in the monomer, dimeric organization slightly differs between the LftR_ΔNtH_ and LftR_CtH_ structures. When the dimeric structures of LftR_ΔNtH_ and LftR_CtH_ were superposed using the wHTH motifs of subunit A, the structures of subunit A were overlaid well, but those of subunit B were not superimposable due to severe positional displacements in the quaternary structures (Fig 5A). The dimer-forming H1ʹ helix and its neighboring wHTHʹ motif of the LftR_ΔNtH_ structure positionally differ from those of the LftR_CtH_ structures with rotation up to ~23° and translation up to ~9 Å. As a result, the inter-wHTH distance of the LftR_CtH_ dimer (~81 Å) is ~11 Å longer than that of the LftR_ΔNtH_ dimer (~70 Å). Given that wHTH transcription factors change their DNA binding affinities by modulating the inter-wHTH distance and the relative orientation of the two wHTH motifs, the intersubunit flexibility of LftR that is observed in the LftR_ΔNtH_ and LftR_CtH_ structures appear to play a key role in the transcriptional regulation of LftR.

### Intersubunit cavity of LftR and its implication in ligand-mediated transcriptional regulation

LmrR, a PadR-2 subfamily member, was structurally characterized to bind a ligand using an intersubunit cavity [17]. To locate the ligand-binding site in the LftR, we searched for a cavity in the LftR dimer structure using the CASTp program [22]. There is a cavity between two subunits in both the LftR_ΔNtH_ and LftR_CtH_ structures (Fig 5B). The intersubunit cavity is surrounded by the H1 and H4 helices from both subunits. The cavity of the LftR_CtH_ dimer structure (cavity surface, ~277 Å^2^; internal volume, ~148 Å^3^) is larger than the cavity of the LftR_ΔNtH_ dimer (cavity surface, ~157 Å^2^; internal volume, ~83 Å^3^) probably due to conformational changes in the dimerization residues. When the structures of LmrR in complexes with the ligands were overlaid on the LftR structure, the ligand molecules of LmrR were colocalized in the LftR cavity, allowing us to propose that the intersubunit cavity of the LftR dimer could be a putative ligand-binding site.

**Fig 6:**
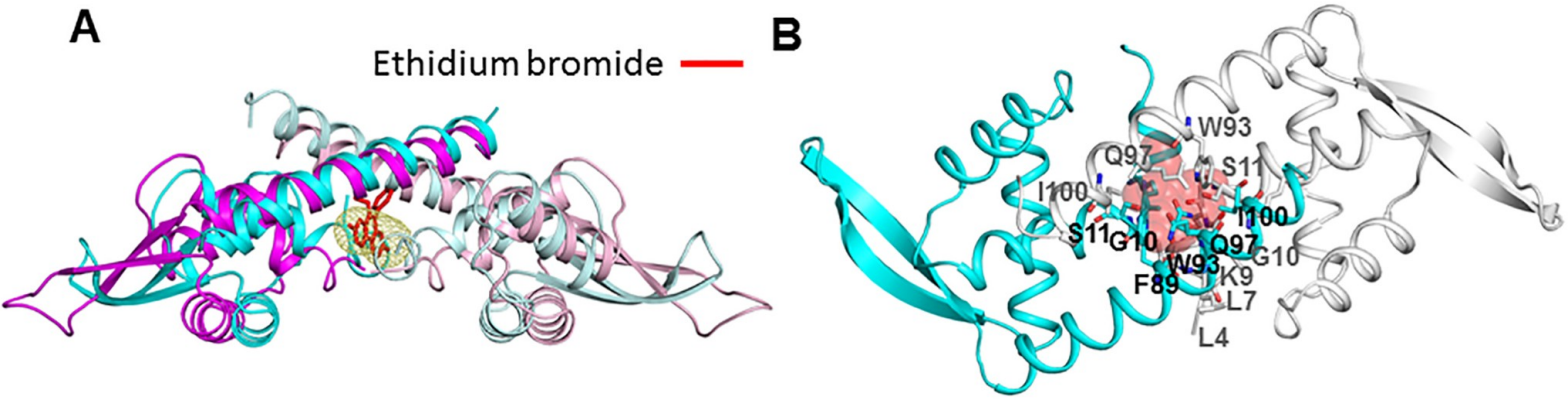
Intersubunit cavity of LftR as a putative ligand-binding site. (A) LftR dimers (the LftR_ΔNtH_ structure, cyan and gray ribbons; the LftR_CtH_ structure, magenta and light magenta ribbons) shown with an ethidium bromide molecule (red sticks), which was identified as an artificial ligand of LftR. (B) Steric clashes of LftR residues with ethidium bromide. Ethidium bromide is shown as red transparent spheres, and the LftR_ΔNtH_ structure is displayed in cyan and gray ribbons. LftR residues involved in steric clashes with the ethidium bromide molecule are shown as sticks with labels.

To provide insight into the ligand-binding and ligand-induced transcriptional regulation mechanisms of LftR, we used an FP assay to analyze the molecular interactions of LftR with ethidium bromide, which was identified as an artificial ligand of LftR [9]. LftR binds ethidium bromide with an equilibrium dissociation constant of 3.70 ± 1.17 μM (S6 Fig). Then, ethidium bromide was placed in the LftR cavity using the AutoDock program [23] (Fig 6 and S7A and S7B Fig). Upon inclusion, the ethidium bromide molecule makes significant clashes with LftR_ΔNtH_ cavity residues (residues 9, 10, 11, 89, 93, 96, 97, 100, 4ʹ, 6ʹ, 7ʹ, 8ʹ, 9ʹ, 11ʹ, 15ʹ, 93ʹ, 97ʹ, and 101ʹ) mainly from the H0, H1, and H4 helices of two subunits (Fig 6B). Therefore, to accommodate a ligand molecule into the LftR_ΔNtH_ cavity, the cavity could be enlarged through conformational changes of cavity residues. Noticeably, the comparative analysis of the LftR_ΔNtH_ and LftR_CtH_ structures indicated that the cavity of LftR is flexible and can be modulated by intersubunit reorganization. Thus, we propose that LftR could rearrange its DNA-binding wHTH motifs along with cavity size and intersubunit organization in response to the ligand in a ligand-mediated transcriptional regulation mechanism.

## Conclusion

The *lftR* gene encodes a *Listeria* protein facilitating invasion that was identified as a transcriptional regulator of a putative multidrug resistance transporter in an opportunistic pathogen, *L*. *monocytogenes*. In this study, we identified two LftR structures and presented the conformational flexibility of LftR, which is essential for ligand-induced transcriptional regulation. A putative operator site of LftR was proposed and its interaction with LftR was experimentally verified by biophysical and biochemical analyses. Our mutational and modeling studies, combined with comparative analyses of the structures and sequences of PadR family members, suggest that LftR recognizes operator DNA in a PadR family-conserved manner as well as through an LftR-specific mode. Moreover, we confirmed that ethidium bromide is an artificial ligand of LftR. Therefore, our study provides in-depth insight into ligand and DNA recognition by the LftR transcriptional regulator. To elucidate the regulatory mechanism of LftR in atomic resolution, formation of a complex of LftR structures with ligand or operator DNA will be required.

## Methods

### Preparation of recombinant LftR protein

The *lmo0719* gene that encodes the full-length LftR protein was amplified by PCR from the genomic DNA of *L*. *monocytogenes* using forward and reverse primers that contain *Bam*HI and *Sal*I restriction enzyme sites, respectively. To prepare a construct that expresses recombinant LftR protein with an N-terminal tag (LftR_NtH_), the PCR product was purified, digested using restriction enzymes, and ligated into a modified pET49b vector that was designed to express recombinant protein with an N-terminal His_6_ tag and a thrombin cleavage site [24, 25]. The resultant plasmid was transformed into *Escherichia coli* DH5α cells. The nucleotide sequence of the LftR_NtH_ expression construct was confirmed by DNA sequencing. For recombinant protein expression, the LftR_NtH_ expression plasmid was transformed into *E*. *coli* strain BL21 (DE3). Cells were grown at 37°C in LB medium containing 50 μg/ml kanamycin. When the optical density of the culture at 600 nm (OD_600_) reached ~0.7, isopropyl β-D-1-thiogalactopyranoside (IPTG) was added at a final concentration of 1 mM to induce recombinant protein expression. The cells were further grown at 37°C for ~3 hours.

After cell harvest by centrifugation, the cell pellet was resuspended in phosphate buffered saline (PBS) solution containing 10 mM imidazole. The cells were homogenized by sonication on ice. The cell lysate was cleared by centrifugation (~25,000 × g). The supernatant was incubated with Ni-NTA resin (Qiagen) at 4°C for 2 hours. The resin was collected by an Econo-Column (Bio-Rad) and washed in PBS containing 10 mM imidazole. The LftR protein was eluted by applying PBS containing 250 mM imidazole. Eluted fractions were analyzed by SDS-PAGE. LftR-containing fractions were dialyzed against a thrombin cleavage-compatible buffer (50 mM Tris, pH 8.0, and 50 mM NaCl). The N-terminal His_6_ tag was removed by thrombin. The resulting tag-free protein (LftR_ΔNtH_) contains six additional amino acids (GSAKDP) at the N-terminus of LftR. LftR_ΔNtH_ was further purified by anion-exchange chromatography using a Mono Q 10/100 GL column (GE Healthcare). The LftR_ΔNtH_ protein was loaded into the column that had been equilibrated in 50 mM Tris, pH 8.0. LftR_ΔNtH_ protein was eluted using a linear NaCl gradient (0.0–1.0 M) at a conductivity of ~16.6 mS·cm^-1^. Fractions were analyzed by SDS-PAGE. Fractions that contained LftR_ΔNtH_ protein were pooled and concentrated to ~18.3 mg/ml.

To eliminate any concern that the presence of additional N-terminal non-LftR residues may affect the intrinsic nature or function of the LftR protein, a LftR expression construct with a C-terminal His_6_ tag (LftR_CtH_) was also generated. The *lmo0719* gene was amplified by PCR from the genomic DNA of *L*. *monocytogenes* using forward and reverse primers that contain *Nde*I and *Xma*I restriction enzyme sites, respectively. The PCR product was digested using *Nde*I and *Xma*I and was ligated to a modified pET49b vector that was designed to express protein with a C-terminal His_6_ tag [24]. The resultant plasmid was transformed into *E*. *coli* DH5α cells. The nucleotide sequence of the LftR_CtH_ expression construct was confirmed by DNA sequencing. For protein expression, the LftR_CtH_ expression plasmid was transformed into *E*. *coli* strain BL21 (DE3). Cells were grown at 37°C in LB medium containing 50 μg/ml kanamycin. When OD_600_ reached ~0.7, IPTG was added to the culture at a final concentration of 1 mM to induce recombinant protein expression. The cells were further grown at 37°C for ~3 hours.

After cell harvest by centrifugation, the cell pellet was resuspended in PBS solution containing 10 mM imidazole. The cells were lysed by sonication on ice. The cell lysate was cleared by centrifugation (~25,000 × g). The supernatant was incubated with Ni-NTA resin (Qiagen) at 4°C for 2 hours. The resin was collected by an Econo-Column (Bio-Rad) and washed using PBS containing 10 mM imidazole. The LftR_CtH_ protein was eluted using PBS containing 250 mM imidazole. The eluted fractions were analyzed by SDS-PAGE. LftR_CtH_-containing fractions were dialyzed against a storage buffer (20 mM HEPES, pH 7.4, and 150 mM NaCl) and concentrated to ~19.7 mg/ml.

Point mutations were introduced into the LftR_CtH_ expression construct using a QuikChange site-directed mutagenesis protocol (Agilent) and DNA primers containing a mutated sequence. The nucleotide sequence of the LftR mutants was confirmed by DNA sequencing. The LftR mutant protein was prepared in a similar manner to the LftR^WT^ protein.

### Gel-filtration chromatography

To analyze the oligomeric state of the purified recombinant LftR protein, gel-filtration chromatography was performed. The LftR protein was loaded on a Superdex 200 10/300 column using a solution containing 20 mM HEPES, pH 7.4, and 150 mM NaCl. Protein elution was monitored by measuring the absorbance at 280 nm. The apparent molecular weight of the eluted protein was estimated based on the elution profile of gel filtration standards (Bio-Rad).

### Crystallization and structure determination

To obtain LftR_ΔNtH_ crystals, 0.5 μl of LftR_ΔNtH_ protein was mixed with 0.5 μl of crystallization solution containing 1 M lithium chloride, 0.1 M citric acid, pH 5.5, and 21% polyethylene glycol 6000. LftR_ΔNtH_ crystals appeared in two days and grew to full size in one week. For X-ray diffraction data collection, the LftR_ΔNtH_ crystals were soaked in a stabilizing solution containing 1 M lithium chloride, 0.1 M citric acid, pH 5.5, and 30% polyethylene glycol 6000. The crystals were directly flash-cooled at -173°C. X-ray diffraction was performed at beamline 7A, Pohang Accelerator Laboratory. The X-ray diffraction data were indexed and scaled using the HKL2000 program [26].

The LftR_CtH_ crystals formed under crystallization conditions with 0.2 M calcium acetate, 0.1 M cacodylate acid, pH 6.3, and 48% polyethylene glycol 600. Crystals were directly flash-cooled at -173°C. The X-ray diffraction data of the LftR_CtH_ crystal were collected at beamline 7A of the Pohang Accelerator Laboratory and were processed using the HKL2000 program [26].

Phases for the LftR_ΔNtH_ and LftR_CtH_ structures were calculated by molecular replacement with the Phaser program [27] using a *Bacillus cereus* PadR-2 subfamily member (PDB accession code 4esf) and LftR_ΔNtH_ as search models, respectively. The final structures of LftR_ΔNtH_ and LftR_CtH_ were generated through iterative cycles of model building and refinement using the Coot [28] and Refman5 programs [29], respectively.

### Native PAGE assay

To describe the interaction of LftR with operator DNA, native PAGE analysis was used [30]. Purified LftR protein (3 μg, ~240 pmol) was incubated with operator DNA (120 pmol, 240 pmol, and 480 pmol) at various molar ratios for 30 min at room temperature. The protein-DNA mixtures were loaded on a native gel, and electrophoresis was performed in running buffer (pH 8.8) containing 25 mM Tris and 190 mM glycine. Protein bands were visualized via Coomassie staining.

### FP assay

To analyze the interaction of LftR with the putative operator DNA, an FP assay [31] was performed. The 28-mer dsDNA containing the OP-1 sequence was labeled with fluorescein at the 5ʹ end of the top strand. As a negative control, bovine serum albumin was used. Fluorescence polarization changes were monitored using an Infinite F200 PRO instrument (Tecan; excitation wavelength, 485 nm; emission wavelength, 535 nm). An equilibrium disassociation constant was determined using the Prism 5 program (GraphPad) based on a single-binding site model.

## Supporting information

S1 Fig Recombinant LftR_ΔNtH_ and LftR_CtH_ proteins used in this study. (PDF)

S2 Fig 2Fo-Fc electron density map of LftR_CtH_. (PDF)

S3 Fig Uncropped image of Fig 3D. (PDF)

S4 Fig Homology-based structural model. (PDF)

S5 Fig Gel-filtration chromatography analysis of LftR proteins. (PDF)

S6 Fig Fluorescence polarization assay of LftR and ethidium bromide binding. (PDF)

S7 Fig Structures of LmrR-ligand complexes. (PDF)

S1 Table Crystallographic statistics of the LftR structures. (PDF)

S1 File 6ABQ_val-report-full. PDB Summary Validation Reports of LftR_ΔNtH_ crystal structure.
(PDF)

S2 File 6ABT_val-report-full. PDB Summary Validation Reports of LftR_CtH_ crystal structure.
(PDF)
