# Supplementary material for: Structure-based molecular characterization and regulatory mechanism of the LftR transcription factor from Listeria monocytogenes: Conformational flexibilities and a ligand-induced regulatory mechanism
Source: PLoS One. 2019 Apr 10;14(4):e0215017. doi: 10.1371/journal.pone.0215017 (PMC6457526; doi:10.1371/journal.pone.0215017)
Supplement: S1 Fig — (PDF) [file pone.0215017.s001.pdf]

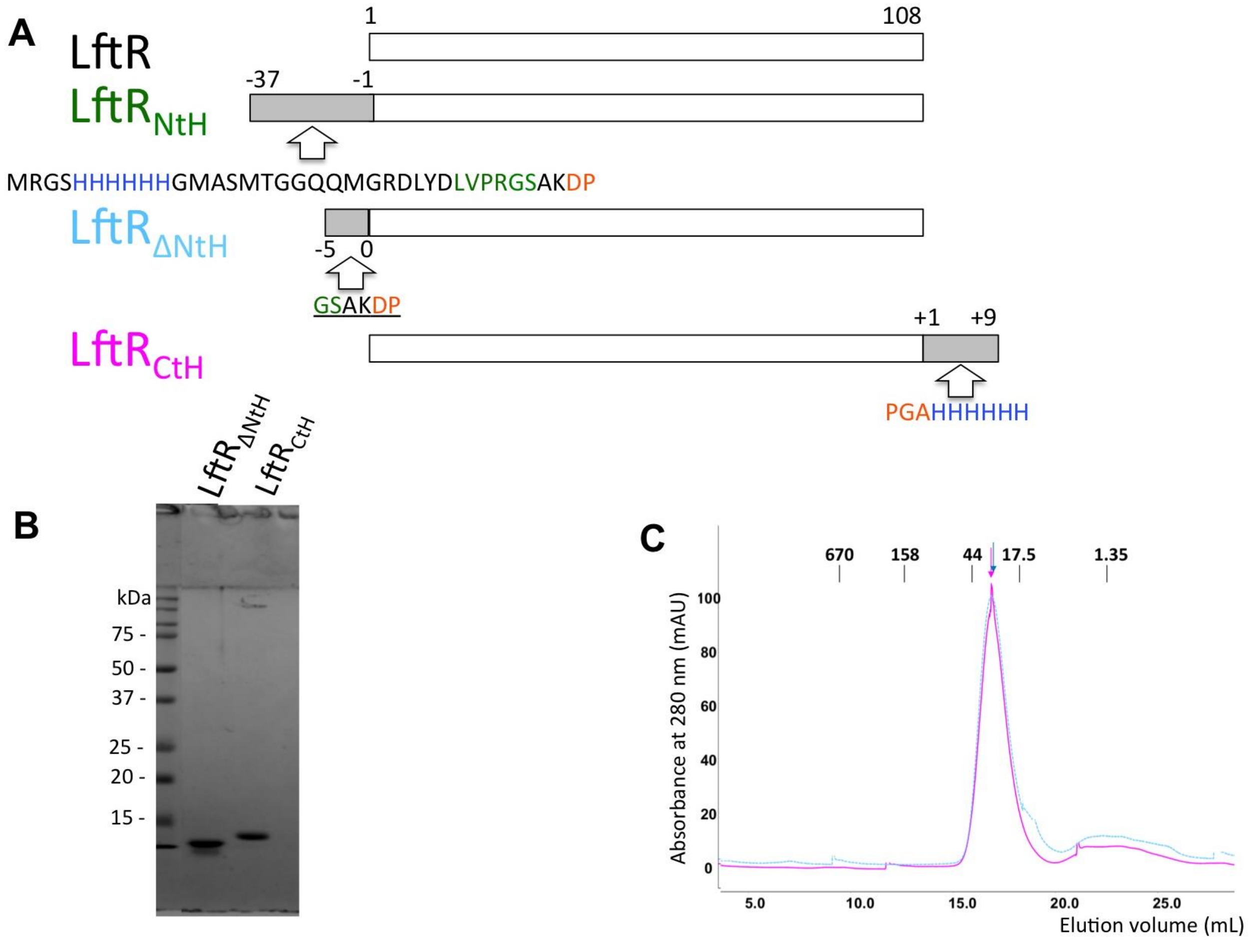

S1 Fig. Recombinant LftR$_{ΔNtH}$ and LftR$_{CtH}$ proteins used in this study. (A) Schematic representations of expression constructs for recombinant LftR proteins. The non-LftR sequences that are appended to LftR in the expression constructs are listed and shown as gray blocks. The non-LftR residues in the LftR$_{ΔNtH}$ structure are underlined. (B) SDS-PAGE analysis of purified LftR$_{ΔNtH}$ and LftR$_{CtH}$ proteins. Protein ladders are shown in the far-left lane of the gel with their molecular weights. (C) Gel-filtration profiles of purified LftR$_{ΔNtH}$ and LftR$_{CtH}$ proteins. Elution peaks of the LftR$_{ΔNtH}$ and LftR$_{CtH}$ proteins were observed at 16.8 ml and 17.3 ml, respectively, and their apparent molecular weights were estimated to be ~24 and ~20 kDa, respectively, based on the elution volumes of gel-filtration standards.
