# Supplementary material for: Structure-based molecular characterization and regulatory mechanism of the LftR transcription factor from Listeria monocytogenes: Conformational flexibilities and a ligand-induced regulatory mechanism
Source: PLoS One. 2019 Apr 10;14(4):e0215017. doi: 10.1371/journal.pone.0215017 (PMC6457526; doi:10.1371/journal.pone.0215017)
Supplement: S2 Fig — (PDF) [file pone.0215017.s002.pdf]

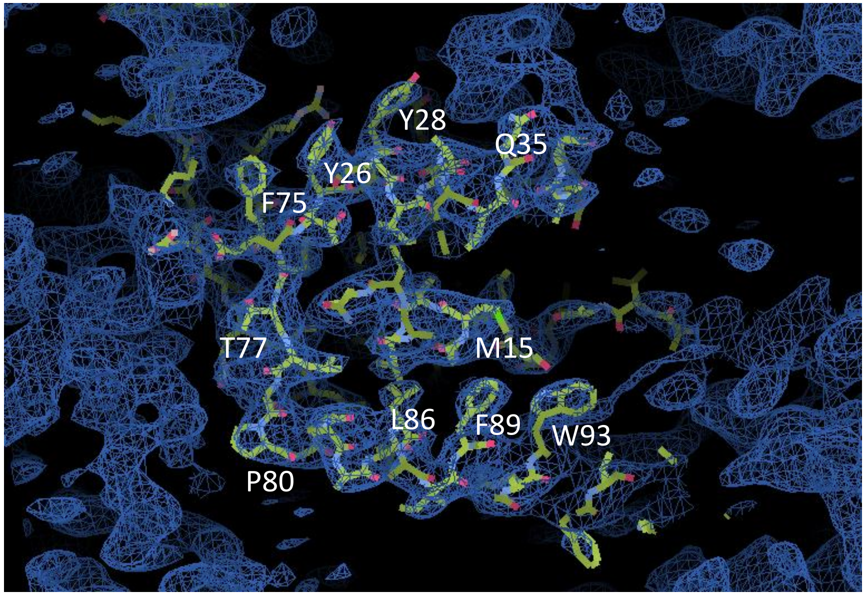

S2 Fig. A 2Fo-Fc electron density map of $LftR_{CtH}$ (contour level of 1.0 σ) at 2.8 Å resolution. The quality of the 2Fo-Fc electron density map (blue mesh) was sufficient to show side chains and matched the atomic model of $LftR_{CtH}$ (sticks: carbon, yellow; oxygen, red; sulfur, green). Some LftR residues are labeled.
