# Supplementary material for: Structure-based molecular characterization and regulatory mechanism of the LftR transcription factor from Listeria monocytogenes: Conformational flexibilities and a ligand-induced regulatory mechanism
Source: PLoS One. 2019 Apr 10;14(4):e0215017. doi: 10.1371/journal.pone.0215017 (PMC6457526; doi:10.1371/journal.pone.0215017)
Supplement: S3 Fig — (PDF) [file pone.0215017.s003.pdf]

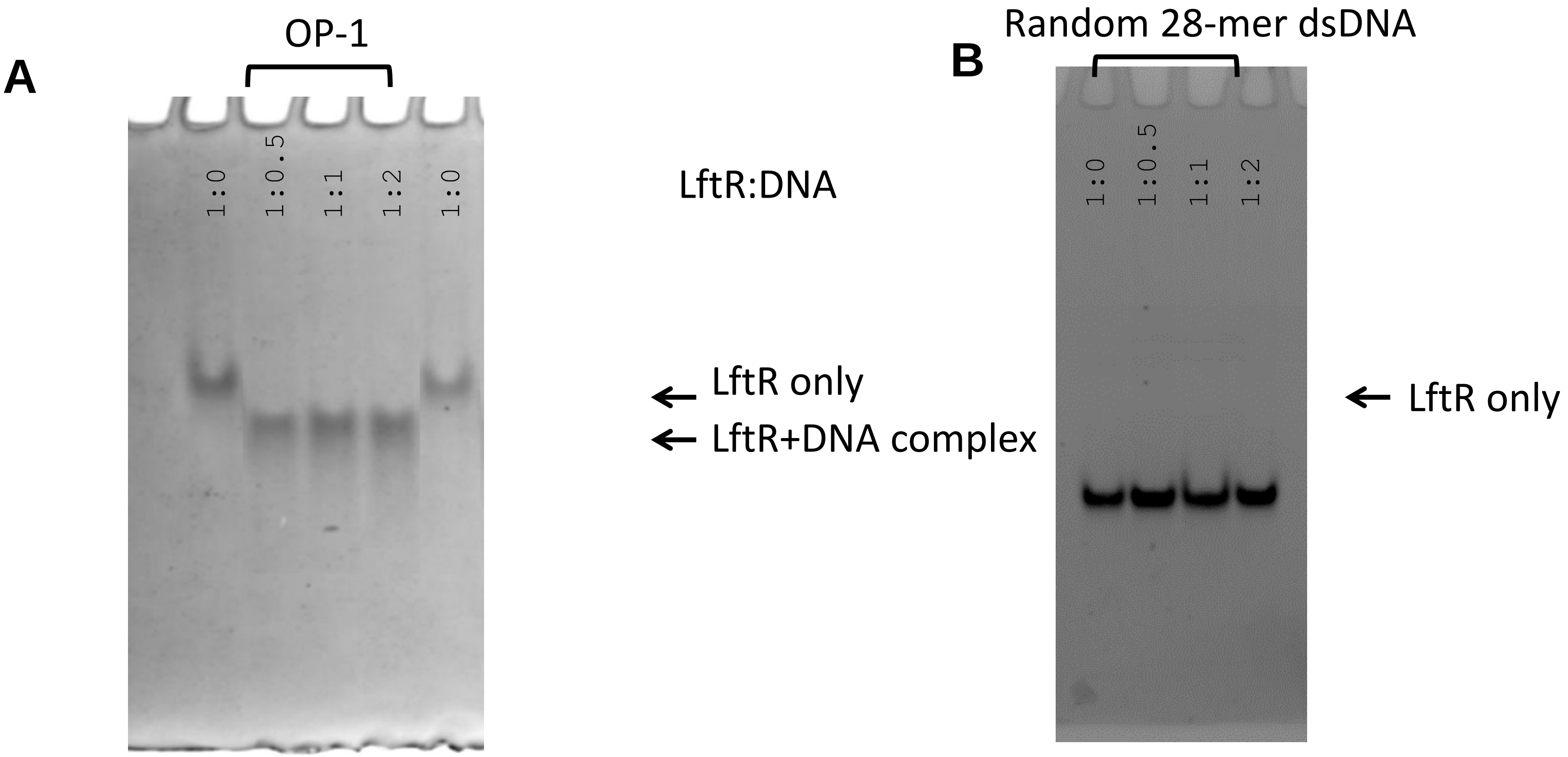

S3 Fig. (A) Uncropped image of Fig. 3D. (B) Native PAGE analysis of LftR protein with random 28-mer dsDNA (5'-CAATCTGTACGGGAACTTTTTATATCAT).
