# Supplementary material for: Structure-based molecular characterization and regulatory mechanism of the LftR transcription factor from Listeria monocytogenes: Conformational flexibilities and a ligand-induced regulatory mechanism
Source: PLoS One. 2019 Apr 10;14(4):e0215017. doi: 10.1371/journal.pone.0215017 (PMC6457526; doi:10.1371/journal.pone.0215017)
Supplement: S4 Fig — (PDF) [file pone.0215017.s004.pdf]

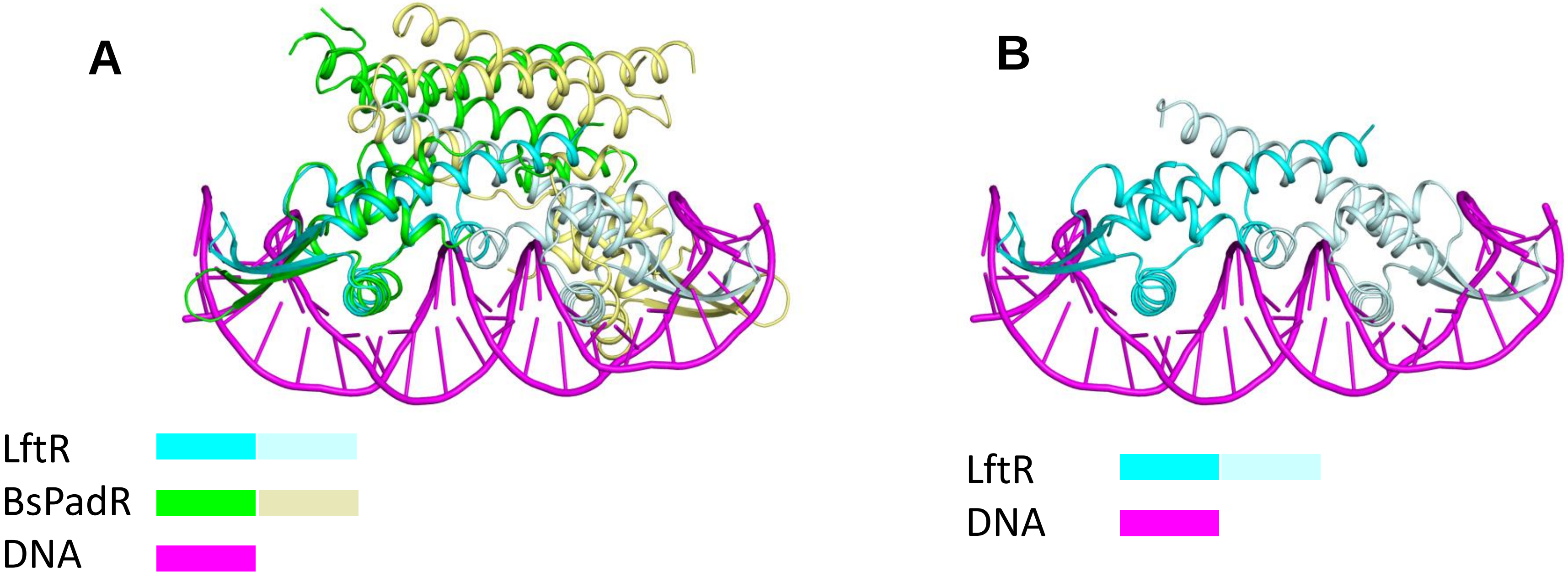

S4 Fig. Homology-based structural model of the LftR-DNA complex. (A) Overlay of the LftR and BsPadR-DNA structures. (B) LftR-DNA complex model shown in ribbons.
