# Supplementary material for: Structure-based molecular characterization and regulatory mechanism of the LftR transcription factor from Listeria monocytogenes: Conformational flexibilities and a ligand-induced regulatory mechanism
Source: PLoS One. 2019 Apr 10;14(4):e0215017. doi: 10.1371/journal.pone.0215017 (PMC6457526; doi:10.1371/journal.pone.0215017)
Supplement: S5 Fig — (PDF) [file pone.0215017.s005.pdf]

A

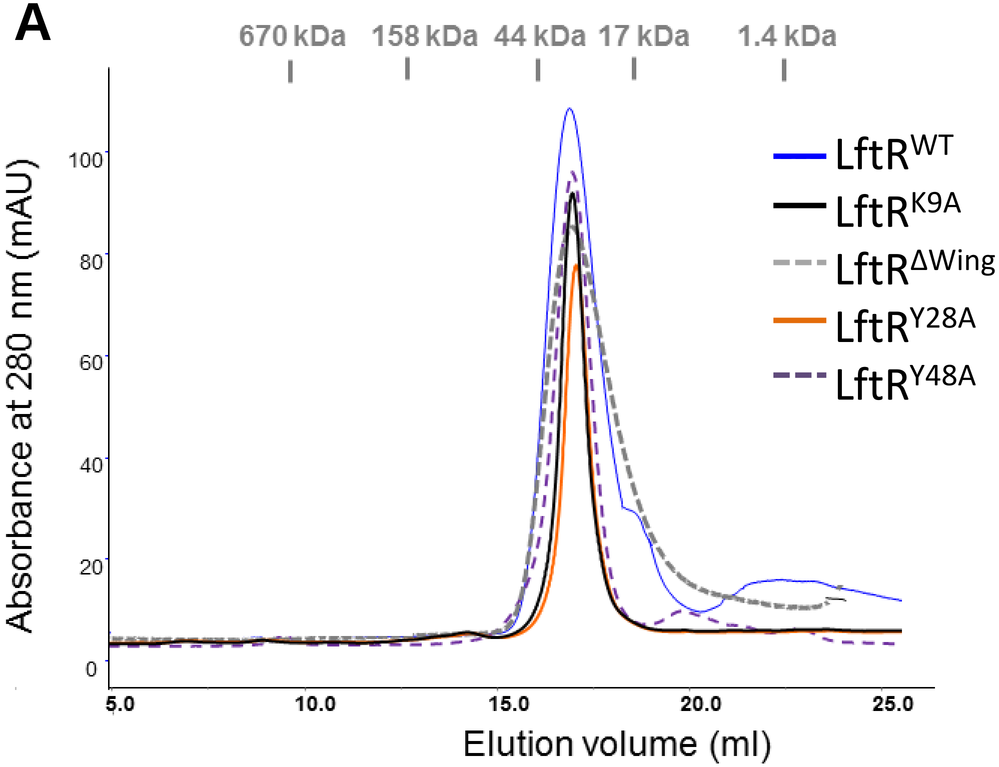


B

| LftR | Elution volume (ml) | Estimated molecular weight (kDa) |
|---|---|---|
| LftR$^{WT}$ | 16.89 | 23.5 |
| LftR$^{K9A}$ | 16.90 | 23.4 |
| LftR$^{R53A}$ | 16.60 | 24.1 |
| LftR$^{K56A}$ | 16.85 | 23.8 |
| LftR$^{\Delta Wing}$ | 16.85 | 23.8 |
| LftR$^{L69A}$ | 16.89 | 23.5 |
| LftR$^{Y28A}$ | 16.92 | 23.0 |
| LftR$^{Y48A}$ | 16.90 | 23.4 |

**S5 Fig.** Gel-filtration chromatography analysis of the LftR wild type and mutant proteins. (A) Elution profiles of LftR proteins and molecular standards (vertical lines). (B) Elution peaks and estimated molecular sizes of the LftR proteins used in Figure 4. The apparent molecular sizes of the LftR proteins were estimated using a standard curve obtained by linear regression analysis of the elution volumes of standards versus log values of molecular weights ($R^2$=0.99). The apparent molecular size of LftR$^{WT}$ was estimated to be ~23.5 kDa. Therefore, we can conclude that LftR forms a dimer because the theoretical molecular weight of an LftR$^{WT}$ monomer is 12.3 kDa. As in LftR$^{WT}$, all of the LftR mutants that we used for our binding study exist in a dimeric form in solution, suggesting that mutations did not modify protein integrity.
