# Supplementary material for: Structure-based molecular characterization and regulatory mechanism of the LftR transcription factor from Listeria monocytogenes: Conformational flexibilities and a ligand-induced regulatory mechanism
Source: PLoS One. 2019 Apr 10;14(4):e0215017. doi: 10.1371/journal.pone.0215017 (PMC6457526; doi:10.1371/journal.pone.0215017)
Supplement: S6 Fig — (PDF) [file pone.0215017.s006.pdf]

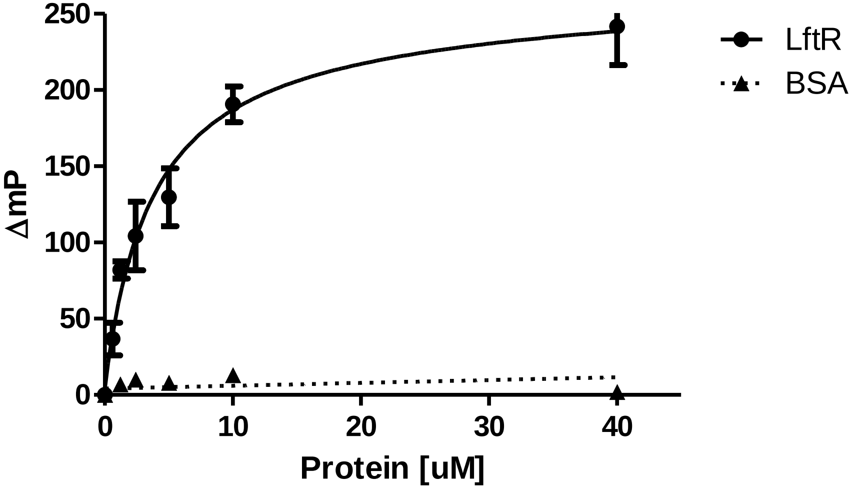

S6 Fig. Fluorescence polarization assay of LftR and ethidium bromide binding. To analyze the interaction of LftR with the artificial ligand, ethidium bromide, an FP assay was performed. The vertical (y) and horizontal (x) axes show increased amounts of milli-polarization of ethidium bromide (ΔmP, excitation wavelength at 485 nm and emission wavelength at 620 nm) and LftR protein (uM), respectively (Left). As a negative control, bovine serum albumin was used. The equilibrium dissociation constant for LftR and ethidium bromide binding was calculated to be 3.70 ± 1.17 uM. The error bars represent the standard deviation for three separate experiments.
