# Supplementary material for: Structure-based molecular characterization and regulatory mechanism of the LftR transcription factor from Listeria monocytogenes: Conformational flexibilities and a ligand-induced regulatory mechanism
Source: PLoS One. 2019 Apr 10;14(4):e0215017. doi: 10.1371/journal.pone.0215017 (PMC6457526; doi:10.1371/journal.pone.0215017)
Supplement: S7 Fig — (PDF) [file pone.0215017.s007.pdf]

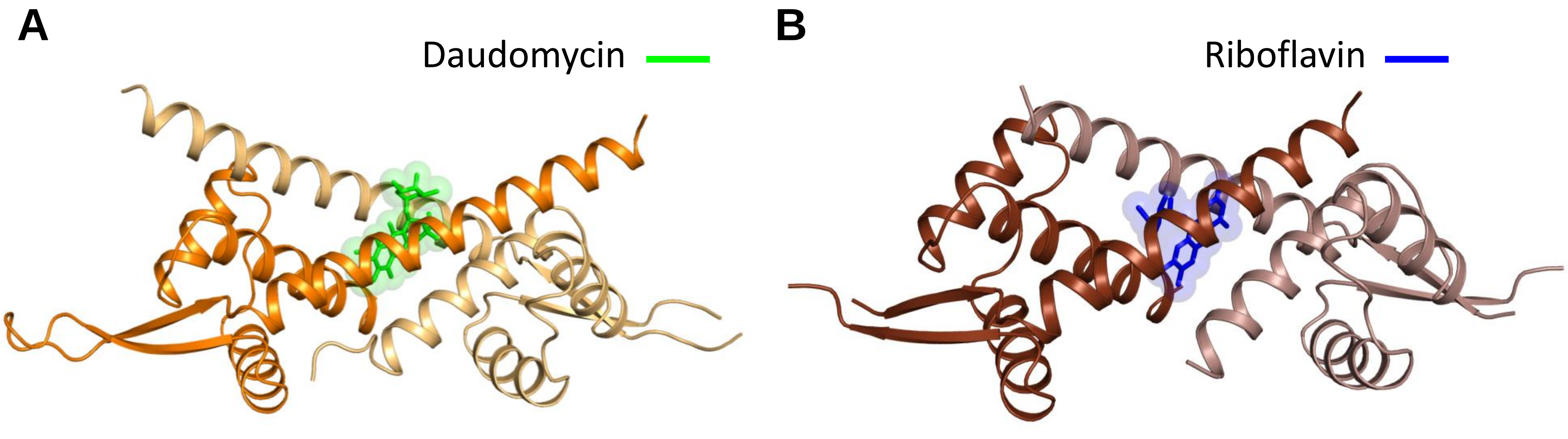

S7 Fig. Structures of LmrR-ligand complexes. (A) Structure of a complex between LmrR (orange and light orange ribbons) and daunomycin (green sticks and green transparent spheres) (PDB accession code 3f8f). (B) Structure of a complex between LmrR (brown and light brown ribbons) and riboflavin (blue stick and blue transparent spheres) (PDB accession code 4zzd).
