# Supplementary material for: Structure-based molecular characterization and regulatory mechanism of the LftR transcription factor from Listeria monocytogenes: Conformational flexibilities and a ligand-induced regulatory mechanism
Source: PLoS One. 2019 Apr 10;14(4):e0215017. doi: 10.1371/journal.pone.0215017 (PMC6457526; doi:10.1371/journal.pone.0215017)
Supplement: S1 Table — (PDF) [file pone.0215017.s008.pdf]

S1 Table. Crystallographic statistics of LftR structures

	$LftR_{\Delta NtH}$	$LftR_{CtH}$
**Data collection**		
Space group	$P3_221$	$P6_4$
Cell parameters		
a (Å)	73.95	67.02
b (Å)	73.95	67.02
c (Å)	66.31	90.29
Wavelength (Å)	1.0003	1.0003
Resolution (Å)	30.00 – 2.20	30.00 – 2.80
Highest resolution (Å)	2.24 – 2.20	2.83 – 2.80
No. observations	77,998	41,461
No. unique reflections	10,969	5,721
R_{merge} (%)[a]	5.3 (14.5)[b]	6.4 (17.6)[b]
I/sigma(I)	49.1 (10.5)[b]	41.3 (8.9)[b]
Completeness (%)	99.7 (89.0)[b]	99.9 (100)[b]
Redundancy	7.2 (5.5)[b]	7.3(7.3)[b]
**Refinement**		
Resolution (Å)	30.00 – 2.30	30.00 - 2.80
No. of reflections (work)	9,111	5,406
No. of reflections (test)	486	295
R_{work} (%)[c]	20.5	25.5
R_{free} (%)[d]	25.3	28.8
No. atoms		
Protein	1,774	1,523
Ligands (Chloride)	3	0
Water	129	4
Average B-value ($Å^2$)	25.0	81.0
RMSD bonds (Å)	0.008	0.010
RMSD angles (°)	1.31	1.53
Ramachandran[e] (favored)	99.3%	99.2%
(outliers)	0.0%	0.0%
PDB ID	6ABQ	6ABT

[a] $R_{merge} = \Sigma_{hkl}\Sigma_i \mid I_i(hkl) - \langle I(hkl)\rangle \mid / \Sigma_{hkl}\Sigma_i I_i(hkl)$

[b] Numbers in parenthesis were calculated from data of the highest resolution shell.

[c] $R_{work} = \Sigma \mid |F_{obs}| - |F_{calc}| \mid / \Sigma |F_{obs}|$ where F_{calc} and F_{obs} are the calculated and observed structure factor amplitudes, respectively.

[d] R_{free} = as for R_{work}, but for 5 % of the total reflections chosen at random and omitted from refinement.

[e] Calculated using MolProbity (http://molprobity.biochem.duke.edu).
