# Supplementary material for: Structure-based molecular characterization and regulatory mechanism of the LftR transcription factor from Listeria monocytogenes: Conformational flexibilities and a ligand-induced regulatory mechanism
Source: PLoS One. 2019 Apr 10;14(4):e0215017. doi: 10.1371/journal.pone.0215017 (PMC6457526; doi:10.1371/journal.pone.0215017)
Supplement: S2 File — PDB Summary Validation Reports of LftRCtH crystal structure. (PDF) [file pone.0215017.s010.pdf]

Full wwPDB X-ray Structure Validation Report (i)

Jul 24, 2018 – 12:51 PM JST

PDB ID	:	6ABT
Title	:	Crystal structure of transcription factor from Listeria monocytogenes
Deposited on	:	2018-07-23
Resolution	:	2.80 Å(reported)

This is a Full wwPDB X-ray Structure Validation Report.

This report is produced by the wwPDB biocuration pipeline after annotation of the structure.

We welcome your comments at *validation@mail.wwpdb.org*
A user guide is available at
https://www.wwpdb.org/validation/2017/XrayValidationReportHelp
with specific help available everywhere you see the (i) symbol.

CONFIDENTIAL VALIDATION REPORT

The following versions of software and data (see references (i)) were used in the production of this report:

MolProbity	:	4.02b-467
Xtriage (Phenix)	:	1.13
EDS	:	rb-20031172
Percentile statistics	:	20171227.v01 (using entries in the PDB archive December 27th 2017)
Refmac	:	5.8.0158
CCP4	:	7.0 (Gargrove)
Ideal geometry (proteins)	:	Engh & Huber (2001)
Ideal geometry (DNA, RNA)	:	Parkinson et al. (1996)
Validation Pipeline (wwPDB-VP)	:	rb-20031172

1 Overall quality at a glance (i)

The following experimental techniques were used to determine the structure:
X-RAY DIFFRACTION

The reported resolution of this entry is 2.80 Å.

Percentile scores (ranging between 0-100) for global validation metrics of the entry are shown in the following graphic. The table shows the number of entries on which the scores are based.

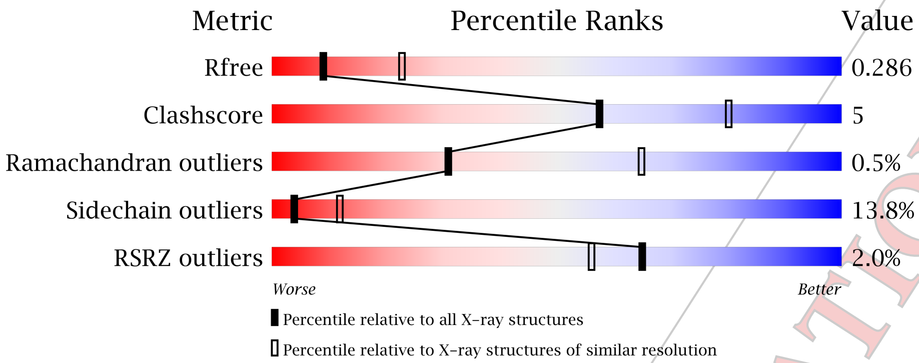

Metric	Whole archive (#Entries)	Similar resolution (#Entries, resolution range(Å))
R_{free}	111664	2792 (2.80-2.80)
Clashscore	122126	3209 (2.80-2.80)
Ramachandran outliers	120053	3158 (2.80-2.80)
Sidechain outliers	120020	3160 (2.80-2.80)
RSRZ outliers	108989	2726 (2.80-2.80)

The table below summarises the geometric issues observed across the polymeric chains and their fit to the electron density. The red, orange, yellow and green segments on the lower bar indicate the fraction of residues that contain outliers for >=3, 2, 1 and 0 types of geometric quality criteria. A grey segment represents the fraction of residues that are not modelled. The numeric value for each fraction is indicated below the corresponding segment, with a dot representing fractions <=5% The upper red bar (where present) indicates the fraction of residues that have poor fit to the electron density. The numeric value is given above the bar.

Mol	Chain	Length	Quality of chain
1	A	98	3% / 76% 21% •
1	B	98	.% / 79% 17% •

CONFIDENTIAL VALIDATION REPORT

2 Entry composition (i)

There are 2 unique types of molecules in this entry. The entry contains 1527 atoms, of which 0 are hydrogens and 0 are deuteriums.

In the tables below, the ZeroOcc column contains the number of atoms modelled with zero occupancy, the AltConf column contains the number of residues with at least one atom in alternate conformation and the Trace column contains the number of residues modelled with at most 2 atoms.

- Molecule 1 is a protein called PadR family transcriptional regulator.

Mol	Chain	Residues	Atoms					ZeroOcc	AltConf	Trace
1	B	98	Total 760	C 496	N 119	O 143	S 2	0	0	0
1	A	98	Total 763	C 498	N 124	O 139	S 2	0	0	0

There are 2 discrepancies between the modelled and reference sequences:

Chain	Residue	Modelled	Actual	Comment	Reference
B	7	ALA	-	expression tag	UNP L8DXR9
A	7	ALA	-	expression tag	UNP L8DXR9

- Molecule 2 is water.

Mol	Chain	Residues	Atoms		ZeroOcc	AltConf
2	B	1	Total 1	O 1	0	0
2	A	3	Total 3	O 3	0	0

CONFIDENTIAL VALIDATION REPORT

3 Residue-property plots (i)

These plots are drawn for all protein, RNA and DNA chains in the entry. The first graphic for a chain summarises the proportions of the various outlier classes displayed in the second graphic. The second graphic shows the sequence view annotated by issues in geometryand electron density. Residues are color-coded according to the number of geometric quality criteria for which they contain at least one outlier: green = 0, yellow = 1, orange = 2 and red = 3 or more. A red dot above a residue indicates a poor fit to the electron density (RSRZ > 2). Stretches of 2 or more consecutive residues without any outlier are shown as a green connector. Residues present in the sample, but not in the model, are shown in grey.

• Molecule 1: PadR family transcriptional regulator

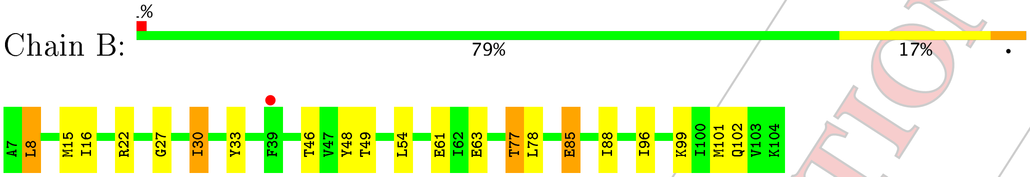

• Molecule 1: PadR family transcriptional regulator

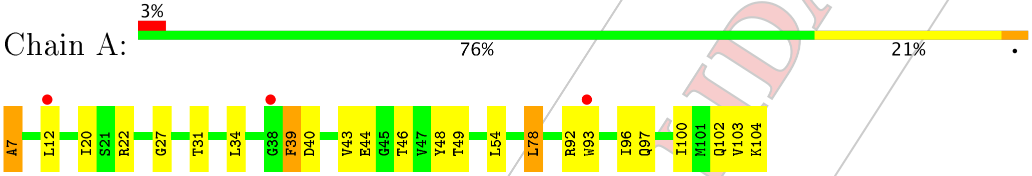

CONFIDENTIAL VALIDATION REPORT

4 Data and refinement statistics (i)

Property	Value	Source
Space group	P 64	Depositor
Cell constants<br>a, b, c, α, β, γ	67.02Å 67.02Å 90.29Å<br>90.00° 90.00° 120.00°	Depositor
Resolution (Å)	58.04 – 2.80<br>31.42 – 2.80	Depositor<br>EDS
% Data completeness<br>(in resolution range)	99.8 (58.04-2.80)<br>99.8 (31.42-2.80)	Depositor<br>EDS
R_{merge}	0.08	Depositor
R_{sym}	(Not available)	Depositor
$< I/\sigma(I) >$ [1]	5.88 (at 2.81Å)	Xtriage
Refinement program	REFMAC 5.8.0103	Depositor
R, R_{free}	0.255 , 0.288<br>0.256 , 0.286	Depositor<br>DCC
R_{free} test set	295 reflections (5.17%)	wwPDB-VP
Wilson B-factor (Å^2)	61.2	Xtriage
Anisotropy	0.696	Xtriage
Bulk solvent k_{sol}(e/Å^3), B_{sol}(Å^2)	0.39 , 77.4	EDS
L-test for twinning[2]	$< \|L\| >$ = 0.51, $< L^2 >$ = 0.35	Xtriage
Estimated twinning fraction	0.487 for h,-h-k,-l	Xtriage
F_o,F_c correlation	0.93	EDS
Total number of atoms	1527	wwPDB-VP
Average B, all atoms (Å^2)	81.0	wwPDB-VP

Xtriage's analysis on translational NCS is as follows: *The largest off-origin peak in the Patterson function is 8.83% of the height of the origin peak. No significant pseudotranslation is detected.*

[1] Intensities estimated from amplitudes.
[2] Theoretical values of $< |L| >$, $< L^2 >$ for acentric reflections are 0.5, 0.333 respectively for untwinned datasets, and 0.375, 0.2 for perfectly twinned datasets.

CONFIDENTIAL VALIDATION REPORT

5 Model quality (i)

5.1 Standard geometry (i)

The Z score for a bond length (or angle) is the number of standard deviations the observed value is removed from the expected value. A bond length (or angle) with $|Z| > 5$ is considered an outlier worth inspection. RMSZ is the root-mean-square of all Z scores of the bond lengths (or angles).

Mol	Chain	Bond lengths		Bond angles	
		RMSZ	#$|Z|$ >5	RMSZ	#$|Z|$ >5
1	A	2.06	1/778 (0.1%)	0.81	1/1048 (0.1%)
1	B	0.58	0/775	0.75	1/1047 (0.1%)
All	All	1.51	1/1553 (0.1%)	0.78	2/2095 (0.1%)

All (1) bond length outliers are listed below:

Mol	Chain	Res	Type	Atoms	Z	Observed(Å)	Ideal(Å)
1	A	7	ALA	CA-CB	54.87	2.67	1.52

All (2) bond angle outliers are listed below:

Mol	Chain	Res	Type	Atoms	Z	Observed(^{o})	Ideal(^{o})
1	A	7	ALA	CB-CA-C	10.25	125.47	110.10
1	B	8	LEU	CA-CB-CG	5.08	126.99	115.30

There are no chirality outliers.

There are no planarity outliers.

5.2 Too-close contacts (i)

In the following table, the Non-H and H(model) columns list the number of non-hydrogen atoms and hydrogen atoms in the chain respectively. The H(added) column lists the number of hydrogen atoms added and optimized by MolProbity. The Clashes column lists the number of clashes within the asymmetric unit, whereas Symm-Clashes lists symmetry related clashes.

Mol	Chain	Non-H	H(model)	H(added)	Clashes	Symm-Clashes
1	A	763	0	757	9	1
1	B	760	0	741	9	0
2	A	3	0	0	0	0
2	B	1	0	0	0	0
All	All	1527	0	1498	16	1

CONFIDENTIAL VALIDATION REPORT

The all-atom clashscore is defined as the number of clashes found per 1000 atoms (including hydrogen atoms). The all-atom clashscore for this structure is 5.

All (16) close contacts within the same asymmetric unit are listed below, sorted by their clash magnitude.

Atom-1	Atom-2	Interatomic distance (Å)	Clash overlap (Å)
1:B:16:ILE:HG12	1:B:30:ILE:HD11	1.46	0.97
1:A:7:ALA:CA	1:A:7:ALA:CB	2.67	0.72
1:A:20:ILE:HG22	1:A:78:LEU:HD22	1.80	0.63
1:B:16:ILE:HG12	1:B:30:ILE:CD1	2.25	0.62
1:B:85:GLU:HA	1:B:88:ILE:HD12	1.89	0.55
1:B:99:LYS:HE2	1:B:99:LYS:HA	1.90	0.54
1:B:33:TYR:OH	1:A:103:VAL:HG13	2.12	0.50
1:A:96:ILE:O	1:A:100:ILE:HG12	2.16	0.45
1:B:27:GLY:HA3	1:B:48:TYR:CZ	2.52	0.45
1:B:15:MET:SD	1:A:103:VAL:HG11	2.56	0.44
1:A:93:TRP:HE3	1:A:97:GLN:HG3	1.84	0.43
1:A:31:THR:HG21	1:A:44:GLU:HA	2.00	0.42
1:A:34:LEU:HD22	1:A:39:PHE:HE1	1.84	0.42
1:B:46:THR:HA	1:B:49:THR:HG22	2.01	0.42
1:A:27:GLY:HA3	1:A:48:TYR:CZ	2.56	0.41
1:B:61:GLU:HB2	1:B:77:THR:HG22	2.03	0.40

All (1) symmetry-related close contacts are listed below. The label for Atom-2 includes the symmetry operator and encoded unit-cell translations to be applied.

Atom-1	Atom-2	Interatomic distance (Å)	Clash overlap (Å)
1:A:104:LYS:NZ	1:A:104:LYS:NZ[4_445]	2.06	0.14

5.3 Torsion angles (i)

5.3.1 Protein backbone (i)

In the following table, the Percentiles column shows the percent Ramachandran outliers of the chain as a percentile score with respect to all X-ray entries followed by that with respect to entries of similar resolution.

The Analysed column shows the number of residues for which the backbone conformation was analysed, and the total number of residues.

Mol	Chain	Analysed	Favoured	Allowed	Outliers	Percentiles	
1	A	96/98 (98%)	92 (96%)	3 (3%)	1 (1%)	17	48

Continued on next page...

CONFIDENTIAL VALIDATION REPORT

Continued from previous page...

Mol	Chain	Analysed	Favoured	Allowed	Outliers	Percentiles	
1	B	96/98 (98%)	93 (97%)	3 (3%)	0	100	100
All	All	192/196 (98%)	185 (96%)	6 (3%)	1 (0%)	31	65

All (1) Ramachandran outliers are listed below:

Mol	Chain	Res	Type
1	A	46	THR

5.3.2 Protein sidechains (i)

In the following table, the Percentiles column shows the percent sidechain outliers of the chain as a percentile score with respect to all X-ray entries followed by that with respect to entries of similar resolution.

The Analysed column shows the number of residues for which the sidechain conformation was analysed, and the total number of residues.

Mol	Chain	Analysed	Rotameric	Outliers	Percentiles	
1	A	76/85 (89%)	66 (87%)	10 (13%)	4	13
1	B	76/85 (89%)	65 (86%)	11 (14%)	3	10
All	All	152/170 (89%)	131 (86%)	21 (14%)	4	12

All (21) residues with a non-rotameric sidechain are listed below:

Mol	Chain	Res	Type
1	B	8	LEU
1	B	22	ARG
1	B	30	ILE
1	B	54	LEU
1	B	63	GLU
1	B	77	THR
1	B	78	LEU
1	B	85	GLU
1	B	96	ILE
1	B	101	MET
1	B	102	GLN
1	A	12	LEU
1	A	22	ARG
1	A	39	PHE
1	A	40	ASP

Continued on next page...

CONFIDENTIAL VALIDATION REPORT

Continued from previous page...

Mol	Chain	Res	Type
1	A	43	VAL
1	A	49	THR
1	A	54	LEU
1	A	78	LEU
1	A	92	ARG
1	A	102	GLN

Some sidechains can be flipped to improve hydrogen bonding and reduce clashes. There are no such sidechains identified.

5.3.3 RNA ⓘ

There are no RNA molecules in this entry.

5.4 Non-standard residues in protein, DNA, RNA chains ⓘ

There are no non-standard protein/DNA/RNA residues in this entry.

5.5 Carbohydrates ⓘ

There are no carbohydrates in this entry.

5.6 Ligand geometry ⓘ

There are no ligands in this entry.

5.7 Other polymers ⓘ

There are no such residues in this entry.

5.8 Polymer linkage issues ⓘ

There are no chain breaks in this entry.

CONFIDENTIAL VALIDATION REPORT

6 Fit of model and data (i)

6.1 Protein, DNA and RNA chains (i)

In the following table, the column labelled '#RSRZ> 2' contains the number (and percentage) of RSRZ outliers, followed by percent RSRZ outliers for the chain as percentile scores relative to all X-ray entries and entries of similar resolution. The OWAB column contains the minimum, median, 95th percentile and maximum values of the occupancy-weighted average B-factor per residue. The column labelled 'Q< 0.9' lists the number of (and percentage) of residues with an average occupancy less than 0.9.

Mol	Chain	Analysed	<RSRZ>	#RSRZ>2			OWAB(Å^2)	Q<0.9
1	A	98/98 (100%)	0.28	3 (3%)	49	38	62, 79, 101, 109	0
1	B	98/98 (100%)	0.24	1 (1%)	82	77	60, 79, 103, 111	0
All	All	196/196 (100%)	0.26	4 (2%)	65	56	60, 79, 103, 111	0

All (4) RSRZ outliers are listed below:

Mol	Chain	Res	Type	RSRZ
1	A	38	GLY	3.7
1	A	12	LEU	2.5
1	A	93	TRP	2.4
1	B	39	PHE	2.2

6.2 Non-standard residues in protein, DNA, RNA chains (i)

There are no non-standard protein/DNA/RNA residues in this entry.

6.3 Carbohydrates (i)

There are no carbohydrates in this entry.

6.4 Ligands (i)

There are no ligands in this entry.

6.5 Other polymers (i)

There are no such residues in this entry.

CONFIDENTIAL VALIDATION REPORT
